# Applications of the Whole-Cell System in the Efficient Biosynthesis of Heme

**DOI:** 10.3390/ijms24098384

**Published:** 2023-05-07

**Authors:** Hongfei Su, Xiaolin Chen, Shijing Chen, Mingzhang Guo, Huilin Liu

**Affiliations:** School of Food and Health, Beijing Technology and Business University, Beijing 100048, China; suhongfei@st.btbu.edu.cn (H.S.); chenxiaolin@st.btbu.edu.cn (X.C.); chenshijing@st.btbu.edu.cn (S.C.); guomingzhang@btbu.edu.cn (M.G.)

**Keywords:** heme, whole-cell manufacturing, metabolic engineering, synthetic biology, cell biosensor

## Abstract

Heme has a variety of functions, from electronic reactions to binding gases, which makes it useful in medical treatments, dietary supplements, and food processing. In recent years, whole-cell system-based heme biosynthesis methods have been continuously explored and optimized as an alternative to the low-yield, lasting, and adverse ecological environment of chemical synthesis methods. This method relies on two biosynthetic pathways of microbial precursor 5-aminolevulinic acid (C4, C5) and three known downstream biosynthetic pathways of heme. This paper reviews the genetic and metabolic engineering strategies for heme production in recent years by optimizing culture conditions and techniques from different microorganisms. Specifically, we summarized and analyzed the possibility of using biosensors to explore new strategies for the biosynthesis of heme from the perspective of synthetic biology, providing a new direction for future exploration.

## 1. Introduction

**Figure 1:**
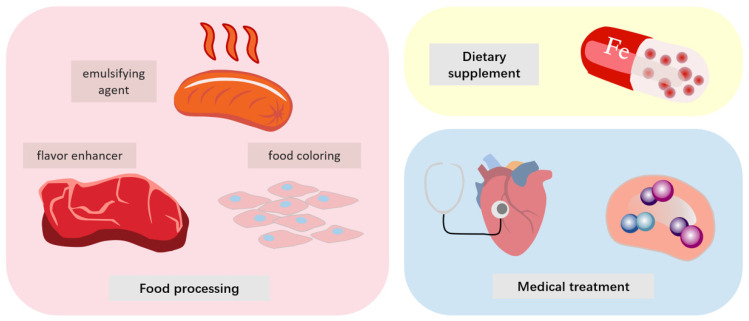
The role of heme in various fields. Common examples are food processing, dietary supplements, and medical fields.

Heme is a complex composed of ferrous ions (Fe^2+^) and protoporphyrin IX (PPIX), a heterocyclic macrocyclic organic compound, forming a heme-Fe^2+^ complex that can bind to O_2_ and CO molecules. In addition, ferric iron (Fe^3+^) is also incorporated into PPIX, resulting in heme-Fe^3+^ complexes that not only bind to NO and SH (SSH) molecules but are often used in the construction of heme sensors. The Lewis acid, hydrophobic, and redox activities of heme enable it to play multiple roles in many biological functions, including catalytic, electron transfer, gas sensing, and transport functions as a cofactor for a variety of proteins, as well as a signaling molecule regulating transcription factors, kinases, ion channels, and cell surface receptors [1,2,3] (Figure 1). For example, heme protein P450s catalyzes ≥ 95% of the reactions known to occur through REDOX chemistry [4]. It can catalyze the specific addition of oxygen atoms on chemical scaffolds. This allows it to play a key role in the production of important molecules using microbial fermentation processes and also in the synthesis of valuable chemicals (e.g., antibiotics, drug metabolites, Steroids and terpenes) have further applications [5], which have given them a leading role in medicine [4]. Furthermore, heme is the most abundant form of iron in mammals, capable of reaching 95% of the total iron quota in the human body, usually 3–4 g of iron [6]. In heme complexes, the redox state of iron can vary, with ferrous PPIX (or heme Fe^2+^ complex) and ferric PPIX (or heme Fe^3+^ complex) being the most common forms [7]. In summary of these properties, heme is gradually gaining widespread use as a bioavailable iron donor in industries such as healthcare and dietary supplements to treat many conditions, including iron deficiency anemia (IDA) in pregnant women [8,9] (Figure 1).

Industrial demand for heme has surged in recent years, primarily due to the significant role heme plays in future foods such as artificial meat. In recent years, 3D printing, shear cell technology, and recombinant protein additives [10,11] used to enhance the sensory properties of plant-based meat (PBM), as well as intensive research in tissue engineering and stem cell biology to produce cell-based meat (CBM) have significantly advanced the field of artificial meat. Heme is an essential flavor catalyst and colorant in animal meat, and adding it to meat analogs has been shown to greatly improve the flavor profile and food-sensory mimicry of these products, making them more appealing to vegetarians and general consumers [10,12] (Figure 1).

Current methods of chemical heme synthesis [13] and free heme isolation [14] from biological samples (e.g., plant tissues and animal blood) using organic extraction are complex, low-yielding, time-consuming, ecologically unfavorable, unable to meet demand, and infringing upon animal welfare [15]. Cell factories based on whole-cell systems provide a new alternative for heme production. Cell factories [16] have the advantages of low cost, high titer, high yield, and high productivity for converting biologically renewable resources into bulk or high-value chemicals, and, therefore, are conceptually similar to what is commonly referred to as a “factory” with “workshops” for different production functions. Their ability to utilize a wide range of carbon sources, from C1 gas to complex biomass, with the potential for cost-effective, sustainable energy production, makes them a promising technology for the environmentally friendly development of renewable resource products to overcome the environmental problems associated with the use of fossil resources [17,18]. The design-build-test-learning (DBTL) cycle in the construction of microbial cell factories has been greatly accelerated [19], and the production of the final product has been greatly facilitated with the advent and development of synthetic biology, systems biology, and metabolic engineering. 

In recent years, heme production has been attempted using cell factories constructed in various strains of bacteria, commonly Escherichia coli [20], Saccharomyces cerevisiae [21], Pichia pastoris [22], and others. Efficient De Novo Biosynthesis of heme within strains based on whole-cell systems is achieved, among other ways, by enhancing the production of the critical precursor 5-aminolevulinic acid (ALA), eliminating competitive pathways [23], and enhancing free heme secretion [24]. In addition, the culture conditions of the strain, such as carbon sources, pH control strategies, iron concentration, induction point, iron content, and cell density, are critical factors for the final heme production. Finally, using whole-cell biosensors in production has a multiplier effect that saves time by enabling high-throughput screening and predicting new genes and pathways to maintain intracellular heme levels. In contrast to the Fe^2+^ complex, the Fe^3+^ complex with sulfate as axial ligand can induce the reaction of most heme sensor proteins under the stabilization of the aerobic atmosphere in reticulocs and erythrocytes, and is therefore often used as a signal for heme sensor [25]. In addition, heme can also be used as a site for sensing gas molecules, which makes heme-based gas sensors (most of which are composed of n-terminal heme iron-binding gas sensitive domain and C-terminal functional domain), based on the structure of the heme–iron complex, can effectively sense gas molecules, such as O_2_, NO, CO, etc. [26,27].

Together with other emerging technologies, such as fluorescence-activated cell sorting (FACS) and next-generation sequencing (NGS) [28], whole-cell biosensors significantly increase heme production and lay the foundation for further exploration. In addition, with the further exploration of the molecular mechanisms of many important reactions involving heme and heme response sensors, clinically meaningful therapeutic targets can be found in a variety of diseases related to ROS, NO, CO, iron, heme biosynthesis and metabolism, and/or bacteria [7, 29]. To lay the foundation for further exploration of strategies for heme production using microorganisms and to indicate potential new directions, this review systematically describes the optimization methods of chassis cells and their culture conditions commonly used for heme production in recent years, the strategies to achieve high heme production based on whole-cell systems, and the application of biosensors in this process.

## 2. Natural Biosynthetic Pathway of Heme

The biosynthesis of heme based on whole cell systems relies heavily on complex regulation in the heme biological pathway in microorganisms. To construct a more optimal heme synthetic pathway, the more genetic elements available in the cell factory, the better, and a comprehensive understanding of the heme synthetic pathway in nature is necessary.

Until 2010, the protoporphyrin-dependent (PPD) pathway was the only known heme biosynthetic pathway, and it was quite prevalent in traditional models of prokaryotes and eukaryotes such as *E. coli*, yeast, plants, and animals [30]. It has been assumed for a long time that all organisms synthesize heme through a similar PPD pathway [31] Unlike eukaryotes, bacteria, and archaea have multiple heme biosynthesis pathways, according to biochemical traces and bioinformatics studies since 1990. Currently, three pathways for heme synthesis are known to exist in nature. The PPD pathway is the most prevalent, but there are two others: the siroheme-dependent (SHD) pathway, the most ancient of the three, and the coproporphyrin-dependent (CPD) pathway, the least common of the three and found only in Gram-positive bacteria [1] Although they share some steps, their entry and exit points and molecular intermediates differ.

### 2.1. Synthesis of ALA

**Figure 2:**
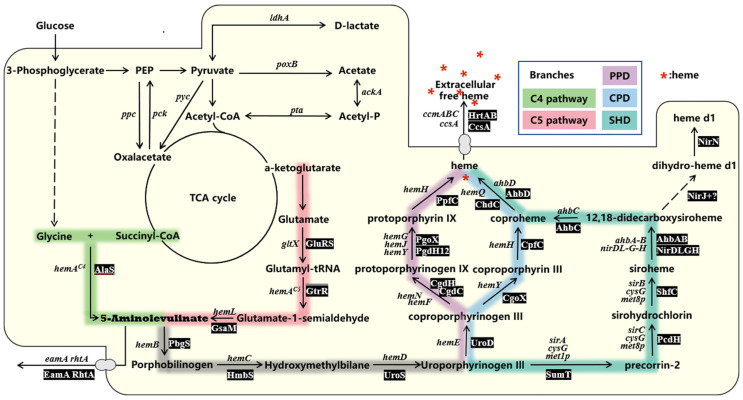
The biosynthetic pathway of heme. The two biosynthetic pathways of 5-aminolevulinic acid (bolded to indicate its importance) are shown in green (C4 pathway) and red (C5 pathway). The three downstream synthetic pathways of heme are marked with blue (CPD), indigo (SHD) and purple (PPD). Solid lines indicate single reactions, and dashed lines indicate more than two reactions. The names of genes encoding the individual enzymes are in italics and some reactions have alternative genes. The abbreviations of the corresponding enzymes are shown in grey rectangle. See Table 1 for a list of names and abbreviations for heme synthesis enzymes.

An essential precursor of heme and the first shared intermediate metabolite in the three heme synthesis pathways described above is ALA (PDD, SHD, CPD). The C4 (“Shemin”) or C5 metabolic pathways are two ways microorganisms can produce them (Figure 2).

The C4 pathway is predominantly found in fungi, α-amastigotes, and postnatal animals such as mammals [15,32], where the pyridoxal-5′-phosphate-dependent enzyme AlaS catalyzes the condensation of glycine and succinyl coenzyme A (CoA) to ALA, accompanied by the release of CO_2_ and coenzyme A as byproducts [33] (Figure 2).

The C5 pathway is the more common pathway of ALA synthesis in archaea, most bacteria, and plants. Compared to the C4 pathway, it is the more efficient and preferred pathway for heme manufacture in the industry due to its ability to obtain higher heme titers with a simple carbon source as a substrate [34,35,36]. In this pathway, there are three steps (Figure 2), glutamyl-tRNA is first obtained by transfer RNA (tRNA) binding to the five-carbon backbone of glutamate catalyzed by glutamyl-tRNA synthetase (*gltX*). Then, glutamyl-tRNA is converted to glutamine-1-semialdehyde catalyzed by glutamyl-tRNA reductase (*hemA*). Finally, it is then catalyzed by glutamate-1-semialdehyde transaminase (*hemL*) to produce ALA [30].

**Table 1:** List of Names and Abbreviations for Heme Synthesis Enzymes and their Encoding Gene in Each Strain.

Abbreviation	Definition	*E. coil*	*Pichia pastoris*	*Saccharomyces cerevisiae*
AlaS	ALA synthase	HemA^C4^	HEM1	HEM1
CgdC	Coproporphyrinogen decarboxylase	HemF	HEM13	HEM13
CgdH	Coproporphyrinogen dehydrogenase	HemN	/	/
CgoX	Coproporphyrinogen oxidase	HemY	/	/
ChdC	Coproheme decarboxylase	HemQ	/	/
ChdH	Coproheme dehydrogenase	/	/	/
CpfC	Coproporphyrin ferrochelatase	HemH	/	/
GluRS	Glutamyl-tRNA synthetase	GltX	/	/
GsaM	Glutamate-1-semialdehyde-2,1-aminomutase	HemL	/	/
GtrR	Glutamyl-tRNA reductase	HemA^C5^	/	/
HmbS	Hydroxymethylbilane synthase	HemC	HEM3	HEM3
PbgS	Porphobilinogen synthase	HemB	HEM2	HEM2
PcdH	Precorrin-2 dehydrogenase	CysG	Met8p	SirC
PgdH1	Protoporphyrinogen dehydrogenase	HemJ	/	/
PgdH2	Protoporphyrinogen dehydrogenase	HemY	/	/
PgoX	Protoporphyrinogen IX oxidase	HemG	HEM14	HEM14
PpfC	Protoporphyrin ferrochelatase	HemH	HEM15	HEM15
ShfC	Sirohydrochlorin ferrochelatase	CysG	Met8p	SirB
SumT	S-adenosyl-L-methionine dependent uroporphyrinogen methyltransferase	CysG	Met1p	SirA
UroD	Uroporphyrinogen decarboxylase	HemE	HEM12	HEM12
UroS	Uroporphyrinogen synthase	HemD	HEM4	HEM4

### 2.2. Formation of Uroporphyrinogen III

The formation of uroporphyrinogen III, the second and last important common intermediate of the three heme downstream biosynthetic pathways, can be separated into three steps (Figure 2). First, a homologous metalloenzyme, porphobilinogen synthase (PbgS), whose catalytic activity depends on metal ions (Zn^2+^ in yeast, bacteria, and mammals or Mg^2+^ in bacteria, and plants) promotes the asymmetric condensation of two ALA molecules to porphobilinogen [37]. Next, hydroxymethylbilane synthase (HmbS) is a monomeric enzyme whose catalytic activity normally depends on the bipyrrole methane cofactor. It catalyzes the condensation of four porphobilinogen molecules to generate hydroxymethylbilane. In the last step, uroporphyrinogen III synthase (UroS) catalyzes the cyclization of hydroxymethylbilane to produce uroporphyrinogen III [38] while promoting the molecular rearrangement of hydroxymethylbilane to avoid the formation of uroporphyrinogen I [39].

### 2.3. Three Downstream Heme Biosynthesis Pathways

The PPD pathway was the first to be discovered, and the related enzymes, and catalytic mechanisms, have been relatively more intensively explored [40,41]. In this pathway, uroporphyrinogen III undergoes a four-step catalytic reaction to obtain heme (Figure 2). First, uroporphyrinogen decarboxylase (UROD) modifies the four acetate side chains of uroporphyrinogen III (decarboxylated) to generate coproporphyrinogen III (CPIII). Next, coproporphyrinogen III decarboxylase (CgdC) (eukaryotes and a few Gram-negative bacteria) or coproporphyrinogen III dehydrogenase (CgdH) (bacteria) catalyze the oxidative decarboxylation of the two propionate substituents on the pyrrole rings A and B of CPIII to the vinyl group of protoporphyrinogen IX [34]. The third step is the catalytic conversion of protoporphyrinogen IX to PPIX. Protoporphyrinogen IX oxidase (PgoX), a member of the FAD-containing superfamily of amine oxidases, acts in eukaryotes and some Gram-negative bacteria, whereas two protoporphyrinogen IX dehydrogenases (PgdH1 and PgdH2) act in most Gram-negative bacteria [42]. In the final step, protoporphyrin ferrochelatase (PpfC) catalyzes the insertion of ferrous iron into the macrocycle of protoporphyrin, allowing the conversion of PPIX to heme [43].

In the CPD pathway (Figure 2), the first step reaction is identical to the PPD pathway and will not be described here (Figure 2). Next, Coproporphyrinogen oxidase (CgoX), which acts similarly to the homologous enzyme (PgoX) in the PPD pathway, removes hydrogen atoms from CPIII through oxygen substitution to produce coproporphyrin III (Figure 2). Interestingly, CgoX can catalyze CPIII while using protoporphyrinogen IX as a substrate [44]. Then, coproporphyrin ferrochelatase (CpfC) catalyzes the insertion of ferrous iron into the scaffold of coproporphyrin III, resulting in coproheme (also a common intermediate in the SHD pathway), a similar step to that catalyzed by PpfC in the PPD pathway (Figure 2). Finally, coproheme decarboxylase (ChdC) decarboxylates the two propionates of the A and B rings of coproheme into vinyl, resulting in heme formation [34] (Figure 2).

Finally, archaea, denitrifying bacteria, and sulfate-reducing bacteria first identified the SHD pathway (Figure 2). It begins with precorin-2 synthesis from uroporphyrinogen III (the final common intermediate of the three-step reaction) commonly used in vitamin B12 synthesis immediately following the conversion of precorin-2 to sirohydrochlorin, which then serves as a substrate to produce sirohaem (Figure 2). Subsequently, the decarboxylation of the acetate side chains on rings C and D, catalyzed by the enzymes AhbA and AhbB, produces 12,18-decarboxylated silicon hemoglobin. It is further removed by the AhbC protein, a deacetylase, from the acetate groups of the A and B rings to produce fecal heme (Figure 2) [45]. Although the final step of the SHD and the CPD pathways are identical, they are catalyzed by two enzymes that have evolved completely independently: AhbD and HemQ, respectively [46]. SHD, the heme *b* synthase AhbD, a monomeric cytoplasmic protein [47], modifies the propionate side chains of the heme A and B rings to vinyl to produce heme.

## 3. Selection of Chassis Cells and Culture Methods

For whole-cell system-based heme production strategies, the final heme yield is highly influenced by the selection of chassis cells and the optimization of culture conditions. Different microorganisms have different background genomes, metabolic environments, and heme tolerance; consequently, the difficulty of constructing heme cell factories and the upper yield limit vary greatly between chassis cells. Chassis cells, the host where synthetic biological reactions occur, are the subject of some operations in genetic and metabolic engineering, and their selection is the basis and key to building an efficient cell factory.

### 3.1. Selection of Strains for Metabolic Regulation

Compared to plant and animal cells, microorganisms are the most suitable chassis cells for heme production through metabolic engineering due to their higher titer and productivity. Common microbial hosts include *E. coli*, *Pichia pastoris*, *Saccharomyces cerevisiae*, *Corynebacterium glutamicum*, *Bacillus subtilis*, and many others.

#### 3.1.1. *Escherichia coli*

In the genome of *E. coli*, there are eight genes related to heme biosynthesis: *hemB*, *hemC*, *hemD*, *hemE*, *hemF*, *hemN*, *hemG*, and *hemH* (Figure 2, Table 1). Due to its extremely low yield, the original strain heme is undetectable by common methods such as High-Performance Liquid Chromatography (HPLC).

According to the findings of Ge et al. [20], upregulation of *hemB* alone or simultaneously with *hemB*, *hemG*, and *hemH* significantly increased heme production. Moreover, increasing the copy number of *hox1* encoding ferredoxin-dependent heme oxygenase (Hox1) and *pcyA* encoding phycocyanobilin: ferredoxin oxidoreductase (PcyA) promoted heme biosynthesis. In addition, Ju et al. [48] co-expressed ALA synthase (*hemA*), NADP-dependent malic enzyme (*maeB*), and dicarboxylic acid transporter (*dctA*) in *E. coli*, producing recombinant *E. coli* capable of producing ALA and heme and serving as a bioavailable iron source. Due to the sophisticated and complex interacting heme production pathways, it is insufficient to regulate the expression of the relevant genes in a general way. To achieve fine regulation, Zhao et al. rechecked the overexpression of genes involved in the C5 pathway of ALA biosynthesis and key genes of downstream heme biosynthesis in plasmids [24] modularly combining the genes involved based on the previous rough regulation. Consequently, the titer of heme within the strain and the amount of free heme secreted at the extracellular level increased significantly. 

#### 3.1.2. Other Common Strains

In *Pichia pastoris*, overexpression of all genes of the heme biosynthetic pathway (HEM1, HEM2, HEM3, HEM4, HEM12, HEM13, HEM14, HEM15) (Table 1) under the control of AOX1p can increase heme levels to some extent [22].

In *Saccharomyces cerevisiae*, there is a natural spatial barrier to heme synthesis. Heme is synthesized in spatially isolated mitochondria and cytoplasm through eight (Hem1p, Hem2p, Hem3p, Hem4p, Hem12p, Hem13p, Hem14p, and Hem15p) enzymatic steps. Moreover, overexpression of *HEM3* could significantly alleviate the rate-limiting effects in the biosynthesis process [21]. In addition, increasing the transcript levels of the *HEM13*, *HEM14*, and *HEM15* genes also improves heme synthesis (Table 1) [49]. However, overexpression of Hem15 may cause oxidative damage and stress in cells due to increased mesoporphyrin [50]. In addition, Xue et al. [49] also demonstrated that Hem14p and Hem15p are efficient in generating heme from the intermediate product (protoporphyrinogen IX) in the cytoplasm and that a truncated assembly of Hem13p with Hem14p and Hem15p can effectively promote heme synthesis, resulting in heme that is sufficient for cell growth.

Diphtheria toxin repressor (DtxR), a global transcriptional regulator in *Corynebacterium glutamicum*, controls the expression of genes encoding membrane proteins and transcriptional regulators involved in iron storage, uptake, and utilization-related transcription. Ko et al. [51] found that iron overload upregulated most heme biosynthetic pathway genes due to DtxR overexpression, which repressed the *hrrA* gene. The activation of HemA^M^ and HemL proteins upregulated the complex and sophisticated heme biosynthetic pathway, increasing heme production in the engineered strain ratio of Growth rate was also restored, increasing total heme production [34].

### 3.2. Optimization of Cultivation Conditions

Over the past 30 years, strategies to improve the titers of ALA and heme by optimizing cultivation processes and investigating relevant medium components have received much attention. 

#### 3.2.1. Cultivation Conditions for ALA Production

ALA is an essential heme precursor, and enhancing its concentration through culture optimization is beneficial for heme production. The common ones are batch replenishment fermentation and two-stage fermentation techniques.

Due to the ionic effect on ALA synthase enzyme activity in normally supplemented cultures [52], the ionic composition of the culture broth plays a crucial role in ALA production during fermentation. Previous studies have demonstrated that Mn^2+^ and Mg^2+^ ion concentrations are crucial factors in *E. coli* ALA production [53]. Recently, Cui et al. [54] demonstrated that 1 g/L MgSO4-7H2O and 0.01 g/L MnSO4-7H2O in the culture medium were suitable for ALA production by *E. coli*. In addition, they also found that yeast extract affected ALA production, and while 12 g/L yeast extract promoted cell growth and ALA accumulation, lower levels significantly affected ALA production. Although yeast extract at 12 g/L promotes cell growth and facilitates ALA accumulation, lower concentrations of yeast extract are more suitable for large-scale industrial organisms production due to their lower cost.

Two-stage fermentation technology refers to two stages of biomass growth decoupling and product synthesis, with the first stage focusing solely on cell growth and the second stage immediately following focusing on product fermentation. The two stages of separation allow cellular resources to focus more on product synthesis, yielding vigorous productivity and higher yields. As a result of the research of Yang et al. [55], non-growing cells were used for two-stage fermentation in ALA production, and the relevant parameters were optimized by a homogeneous design. The cells were transferred to a fermentation buffer with added glucose and glycine after the first stage for the second stage of fermentation, and the yield of ALA obtained was nearly 1.67 times higher than that obtained with *E. coli* cultured by normal supplementation due to the simplicity of the buffer system.

#### 3.2.2. Cultivation Conditions for Heme Production

In the biosynthesis of heme, each vitamin plays a different and irreplaceable role. For example, biotin maintains the level of the medium during gluconeogenesis and the citric acid cycle, nicotinic acid enhances precursor carbon supply, pantothenic acid is a substrate for CoA biosynthesis, and cobalamin is a coenzyme for various enzymatic reactions. Based on this background, Lee et al. [56] investigated the effect of adding major vitamins to the culture medium on heme production. The addition of the previously mentioned vitamins (0.01 mg biotin, 5 mg niacin, 1 mg pantothenic acid, 1.4 mg cobalamin per liter) to a medium containing phosphate buffer and bicarbonate resulted in not only a 2.5-fold increase in the growth rate of the bacteria but also a 2-fold increase in the final heme synthesis.

The final step of heme synthesis requires the participation of Fe^2+^, so supplementing the medium with Fe^2+^ required for the biosynthesis process may enhance the heme production, commonly FeCl_2_ [15] and FeSO_4_ [57]. Zhao et al. [24] used the pH-stat technique to automatically add 200 mg L^−1^ of FeSO_4_∙7H_2_O to the fed solution when the pH surpasses a preset value. Therefore, the intracellular heme titer of recombinant *E. coli* increased from 13.9 ± 0.7 mg L^−1^ to 32.7 ± 0.7 mg L^−1^, and the percentage of heme secreted into the extracellular compartment increased. 

Since the synthesis of a heme molecule requires four nitrogen atoms, the addition of an additional nitrogen source to the medium has a significant effect on the heme yield. The experimental results of Zhao et al. confirmed this conclusion [24]. They added 5 g L^−1^ of (NH_4_)_2_SO_4_ to the supplement solution as an additional nitrogen source. Following fermentation, they found that the intracellular heme titer and the percentage of extracellular heme had increased. For this reason, Ko et al. [51] investigated the effect of adding common nitrogen sources (ammonium sulfate, tryptophan, ammonium chloride, and caseinate hydrolysate) to culture broth on heme production. The medium supplemented with 10 g L^−1^ tryptophan was more effective at producing heme.

Environmental factors also play a significant role in determining the efficiency and amount of heme production during culture. The appropriate temperature is conducive to the growth of the strain. Zhao et al. [24] lowered the culture temperature to 30 ℃, which is lower than the previous incubation temperature of 37 °C [48,58]. Similarly, Ko et al. [51] cultured recombinant *E. coli* at 30 °C with 10 mg/L FeSO_4_∙7H_2_O in the culture medium and obtained similar results.

Suitable chassis cells and culture conditions only play a minor role in using the whole cell system to produce heme, so further research is needed.

## 4. Synthetic Biology Strategies to Enhance Heme Production

**Figure 3:**
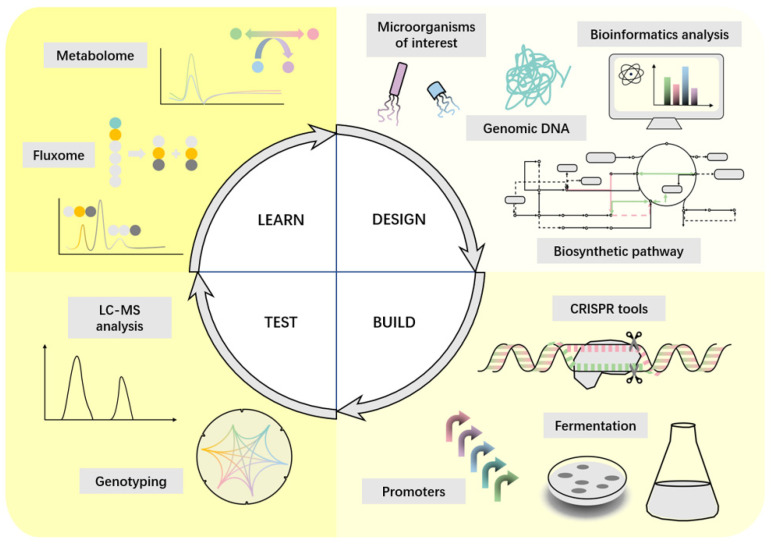
A synthetic biology-inspired approach based on a design, build, test and learn cycle. In the design phase, bioinformatics analysis of whole genome sequences is performed using genome mining computational tools. In the construction phase, genetic engineering tools are used to clone, assemble and reconfigure the desired biosynthetic pathways with genetic elements and circuits. Finally, novel, enhanced and diverse biosynthetic pathway structures with practical applications are screened.

**Figure 4:**
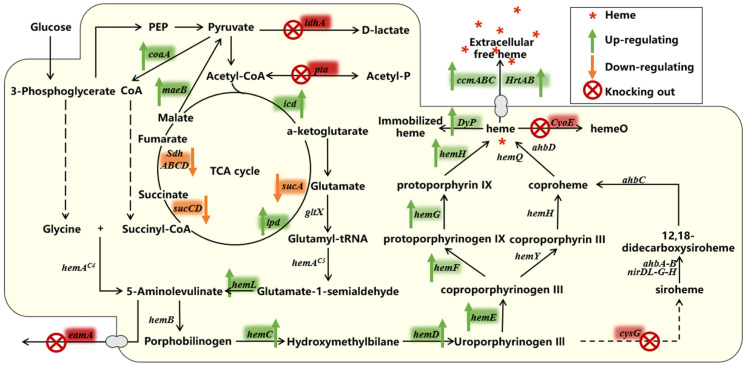
Synthetic biology strategies to enhance heme production. Green, orange, and red color blocks indicate genes that need to be up-regulated, down-regulated, and knocked out, respectively.

The purpose of synthetic biology is to combine biology and engineering to create novel biological functions and systems not found in nature. Synthetic biology develops cell factories by introducing new non-native pathways to produce target compounds in optimized chassis strains [16]. The process usually follows a DBTL cycle. A commonly used optimization process has three steps: (1) direct optimization of the pathway in any possible way; (2) removal of competing activities; (3) application of global regulatory engineering [59]. The process allows for more versatile and standardized options for developing cell factories while accelerating cycle efficiency (Figure 3) by modifying programmable synthetic biology tools [60] such as CRISPR-Cas for different purposes, including efficient genome engineering and modular assembly of complex biological systems with unique functions. Common strategies are shown in Figure 4 and Table 2.

### 4.1. Enhances the Ability to Produce the Important Precursor ALA

As an essential precursor in heme biosynthesis, ALA significantly increases the ability to produce heme, which plays a crucial role.

#### 4.1.1. Supplementation of the Substrate Succinyl CoA

Succinyl CoA is one of the substrates of ALA; therefore, an increase in intracellular CoA may contribute to heme biosynthesis.

Many scholars have attempted to enhance the supply of succinyl coenzyme a by increasing the metabolic flux to the positive tricarboxylic acid (TCA) cycle. Oh-Hee Kwon et al. overexpressed the *maeB* encoding malic enzyme (MAE), which provides the precursor pyruvate for CoA, in *E. coli*. The *coaA* encoding pantothenate kinase (PANK) gene was also overexpressed to provide additional CoA for ALA synthesis, resulting in a 10% increase in ALA production [55] and a twofold increase in the final heme product [48]. Peng et al. [55] directly catalyzed the synthesis reaction of succinyl CoA and increased ALA production by approximately 6% by overexpressing *odhA-sucB-lpd* encoding 2-oxo-tartaric acid dehydrogenase in recombinant *Bacillus glutamicus*. In addition, Ge et al. [61] up-regulated the upstream genes *icd* and *pda* of succinyl CoA in the TCA cycle and down-regulated the downstream genes *sucCD*, which encodes succinyl CoA synthase, *sdhAB*, which encodes succinic dehydrogenase subunits AB, and *sdhCD*, to amplify the source of succinyl CoA, reduce its consumption, and increase the carbon flux to ALA. Subsequently, Ding et al. [62] also knocked out *sucCD* and *sdhAB* to block the conversion of succinyl CoA to other downstream metabolites. The experiment results revealed that only *sucCD* deletion significantly increased ALA titer (1.31 g/L), whereas *sdhAB* knockdown had no effect.

#### 4.1.2. Reduced Secretion of ALA to the Extracellular

In addition, it is also possible to increase ALA titer by decreasing its secretion. 

Most cells still require transporter proteins to accelerate ALA export, although cells can metabolize ALA out of the cell spontaneously. 

EamA is a non-specific amino acid transporter membrane protein [63], a major facilitator superfamily transporter, participating in ALA secretion. Feng et al. [64] knocked down *eamA* while ensuring the cell growth state. A decrease in ALA secretion led to a metabolic flux increase for downstream heme synthesis and a 42.9% increase in intracellular heme content.

In addition to *eamA*, Kang et al. discovered RhtA, an endosomal transporter protein encoded by the *rhtA* gene that effluxes isotopes and threonine with broad substrate specificity and accelerates ALA production in *E. coli* [65]. Following this, RhtA frequently functions as a non-specific transporter and plays a crucial role in the efficient production of ALA by *E. coli* as an extracellular [66]. In addition, many scholars have also introduced *E. coli rhtA* into *Corynebacterium glutamicum* as a more efficient and cost-effective strategy for producing extracellular ALA than *E. coli* [55,67,68]. Conversely, in the future, it may be possible to reduce ALA secretion to the extracellular compartment by down-regulating *rhtA* expression in *E. coli*, thereby enhancing the intracellular ALA titer and favoring heme production.

#### 4.1.3. Regulation in the Heme Biosynthesis Pathway

Multiple genes in the heme biosynthetic pathway may be regulated to affect ALA titers and heme production; this pathway is complex and sophisticated, with meticulous relationships between various intermediate metabolites. Ju et al. [48] found that increased heme downstream synthesis pathway gene expression (*hemC*, *hemD*, *hemE*) could also increase ALA levels. Similarly, Zhang et al. [69] found a significant increase in ALA titer and cell biomass in recombinant *E. coli* overexpressing *hemA, hemL, hemD, and hemF* after 7.5 mg/L addition. These methods all facilitated the achievement of high heme production.

In addition, some metabolic regulatory strategies use heme production pathways to increase ALA titers, which is detrimental to end-product heme production by down-regulation downstream genes. For example, Tianyuan Su et al. [70] found that using CRISPR interference (CRISPRi) to down-regulate HemB expression, which is involved in heme biosynthesis, resulted in a significant increase in ALA accumulation but a decrease in heme production. Experiments conducted by Li et al. [57] demonstrated that sRNA RyhB inhibited the expression of iron-binding proteins and led to the down-regulation of the transcript levels of *hemB* and *hemH*, both increased ALA accumulation by 116%. However, there was a noticeable reduction in heme accumulation downstream of the heme biosynthesis pathway, and cell growth was also affected to some extent. Therefore, this strategy is also not suitable for heme production.

Although ALA as a precursor affects heme production, heme as a final product influences its level in a complex regulatory pathway. Feng et al. [64] heterologously expressed dye-decolorizing peroxidase (DyP), a heme peroxidase from *Thermobifida fusca*, to immobilize heme, thereby reducing free heme levels in cells and alleviating the feedback inhibition of ALA synthesis, which in turn promoted heme production. Similarly, Ge et al. [20] upregulated *hemAs* and *hemL* to promote ALA synthesis while reducing the feedback inhibition caused by heme accumulation, which had a similar effect on final heme production.

#### 4.1.4. Other Regulations in TCA Cycle

Not only does the heme synthesis pathway influence ALA accumulation, but so do engineered metabolic fluxes from TCA cycles and the upstream.

By eliminating *sucA* from *E. coli*, Noh et al. [71] blocked the TCA cycle, accumulating α-ketoglutarate and allowing more carbon flux to flow ALA production, resulting in an 8.77-fold increase in ALA production. Associated intermediates are synthesized through the glyoxalate cycle when the TCA cycle is interrupted. However, excessive carbon flux may result in a shortage of α-ketoglutarate, which is required for ALA production, whereas insufficient carbon flux may impair cell growth due to a lack of essential metabolites such as succinate and oxaloacetate [54]. Consequently, *aceA*, which encodes isocitrate lyase, is crucial for precise carbon flux regulation in the glyoxalate cycle. Engineered *E. coli* with moderate AceA activity, obtained by altering transcriptional intensity, have abundant cellular biomass and high ALA production.

Interestingly, Ge et al. [61] found that in *C. glutamicum*, knockdown aceA directly alleviated competition between the glyoxylate and TCA cycles and increased ALA titers. In addition, they deleted *gdhA*, which encodes glutamate dehydrogenase, to reduce the carbon flux to glutamate, an unwanted metabolite, to increase ALA production.

### 4.2. Removing Competitive Pathways

With limited substrate, deleting the competing pathway allows carbon flux to flow to the target product, resulting in a more efficient production process and improved yields.

To remove the competitive pathway, Geng et al. [15] first knocked out *cysG* encoding sirohydrochlorin ferrochelatase and *hemX* encoding uroporphyrin-III C-methyltransferase in *E. coli JM109(DE3)*, which is involved in extracellular transport of heme production precursors, to block Siroheme synthesis and increase heme titers. In addition, CyoE disruption prevented the conversion of *hemeO*, resulting in a dramatic increase in heme accumulation and production, while ALA production remained almost unchanged. Similarly, in *E. coli*, knockout of PTA, encoding the phosphate acetyltransferase that catalyzes acetate synthesis, and ldhA, encoding the lactate dehydrogenase that catalyzes lactate synthesis, ensured a metabolic flux from glucose to l-glutamate, resulting in a doubling of the amount of succinate produced and an accumulation of pyruvate and some citrate in the culture [72], which is beneficial for heme synthesis. Zhao et al. [24] confirmed that gene regulation in the heme pathway increased intracellular heme from 4.2 ± 0.6 mg L^−1^, an increase over the 0.53 mg L^−1^ produced by control bacteria. In this study, *yfeX*, which encodes a peroxidase YfeX that catalyzes the production of porphyrins from porphobilinogen [23] and whose overexpression may disrupt heme homeostasis [73], was hypothesized to encode a putative heme-degrading enzyme that was also deleted in *E. coli* to enhance heme production.

### 4.3. Enhance the Secretion of Intracellular Free Heme

Previous studies have shown that direct overexpression of key genes in the heme synthesis pathway results in the accumulation of toxic and thus detrimental to cell growth byproducts, including porphyrins and the final heme product. Commonly, the secretion of intracellular free heme into the extracellular compartment is increased.

The general strategy is to enhance the secretory capacity of the membrane heme transporter. Zhao et al. [24] overexpressed a *ccmABC* gene encoding a potential heme exporter in engineered *E. coli* and detected an increase in total heme titer and extracellular heme percentage in the strain after fermentation. Similarly, Geng et al. [15] overexpressed Dpp in recombinant *E. coli* by gene editing techniques, resulting in a 2.08-fold increase in final heme production [15]. In addition, previous studies have demonstrated that cytochrome C exporter (CcsA) and transporter A (HrtA) and B (HrtB) in *C. glutamicum* reduce toxic heme concentrations and play a crucial role in extracellular heme secretion [74]. Experiments conducted by Ko et al. [51] demonstrated that although overexpression of the CcsA protein in *C. glutamicum* increased heme secretion in the strain, heme production and specific growth rate reduced significantly. Therefore, this strategy is inappropriate for heme production. In contrast, strains overexpressing the HrtA and HrtB transporter exhibited a 2.78-fold increase in heme secretion and did not exhibit this detection.

In addition, heme may be consumed during extracellular transport by binding to heme-binding proteins on the membrane. The heme-binding membrane proteins HtaA, HmuT, and HrrS in *C. glutamicum* are essential for heme biosynthesis regulation and secretion into the cytoplasm. Among these are HmuT, which regulates heme import [75], and HrrS, a histidine kinase that, when bound to exogenous heme, regulates heme homeostasis with HrrA, a heme manipulator transcriptional repressor [76]. Therefore, the engineered *C. glutamicum* obtained by Ko et al. [51] after generating absurd mutations in wild-type *C. glutamicum* using the CRISPR-cas12a system to disrupt the genes encoding the above three proteins not only reduced heme binding to membrane proteins but also promoted heme biosynthesis. The strains showed a heme secretion of 6.58 ± 0.63 mg L^−1^ and an even higher yield of 35.65 ± 0.67 mg L^−1^ when HrtA and HrtB transporter overexpression was combined with additional metabolic engineering.

**Table 2:** Biosynthesis of Heme in Microorganisms.

Categories		Microorganisms	Strategies	Ref
Increase the titer of ALA				
	Supplementation of the substrate succinyl CoA	*E. coli*	Overexpressing the maeB encoding MAE and the coaA encoding PANK to provide the precursor pyruvate for CoA and additional CoA for ALA synthesis, respectively	[75]
			Knocking out sucCD to block the conversion of succinyl CoA to other downstream metabolites.	[49]
		*Bacillus glutamicus*	Overexpressing odhA-sucB-lpd encoding 2-oxo-tartaric acid dehydrogenase to catalyze the synthesis reaction of succinyl CoA.	[57]
		*C. glutamicum*	Upregulating the expression of the upstream genes icd and pda of succinyl CoA while down-regulating the expression of the downstream genes sucCD and sdhABCD to amplify the source of succinyl CoA, reduce its consumption	[21]
	Reduced secretion of ALA to the extracellular	*E. coli*	Knocking down eamA to reduce ALA secretion	[72]
	Regulation in the heme biosynthesis pathway	*E. coli*	Heterologously expressing DyP to immobilize heme and alleviate the feedback inhibition of ALA synthesis	[72]
			Enhancing expression of hemC, hemD, hemE in the downstream synthesis pathway of heme	[14,75]
			Upregulating hemAs and hemL to promote ALA synthesis while reducing the feedback inhibition of heme	[12]
	Other regulations in TCA cycle	*E. coli*	Eliminating sucA to block the TCA cycle result in accumulating α-ketoglutarate and thus allowing more carbon flux to flow to ALA production,	[58]
		*C. glutamicum*	Knocking down aceA and deleting gdhA to alleviate competition between the glyoxylate and TCA cycles and reduce the carbon flux to glutamate.	[21]
Removing competitive pathways		*E. coli*	Knocking out CysG and hemX to block the synthesis of Siroheme and knocking out CyoE to prevent conversion of heme to hemeO	[77]
			Knocking out of pta catalyzing acetate synthesis and ldhA, that catalyzing lactate synthesis to ensure a metabolic flux from glucose to l-glutamate	[78]
			Deleting yfeX to prevent disruption of heme homeostasis	[79]
Enhance the secretion of intracellular free Heme		*E. coli*	Upregulating ccmABC encoding a potential heme exporter	[79]
		*C. glutamicum*	Overexpressing the HrtA and HrtB transporter	[54]
			Disrupt the HtaA, HmuT and HrrS by the CRISPR-cas12a system to reduce the binding of heme to membrane proteins	[54]

## 5. Whole-Cell Biosensor in Heme Production

Heme, involved in electron transfer, oxygen storage, and enzymatic reactions, is essential for living organisms. However, excessive free heme concentrations in cells are toxic because they generate large amounts of reactive oxygen species. By causing irreversible damage to nucleic acids, proteins, and oxidized membranes, these reactive oxygen species create a pro-inflammatory environment detrimental to cell growth and heme production. Therefore it is necessary to monitor intracellular hemoglobin in real time [78,80].

Whole-cell biosensors can be used as a low-cost assay for the target instead of analytical instruments using quantifiable signals from reporter proteins to identify bioavailable levels of target analytes in various sample types with high specificity. Similarly, whole-cell biosensors play a crucial role in heme production [81].

### 5.1. Biosensor for ALA Detection

Using cell factories based on whole-cell systems for heme production sometimes involves a modular stepwise optimization strategy, such as optimizing ALA production first. Biosensors for detecting ALA enable high-throughput screening of experimental strains, vastly improving efficiency and allowing direct visual discrimination. As early as 2005, Chung et al. [82] tried to bind the green fluorescent protein gene (*egfp*) to *hemA* and use the green fluorescence intensity to respond to the ALA concentration. Although this enabled the characterization and study of extracellular ALA, the precise value of its concentration still relied on the Ehrlich reagent. On this basis, Tan et al. [83] fused super folder green fluorescent protein (sfGFP) to R. globosporus HemA (*RshemA*), using a dual promoter and dual plasmid, to detect and produce ALA simultaneously. 

The above strategies involve binding fluorescent proteins to genes in the production pathway and indirectly responding to ALA production with the degree of gene transcription. The assumption underlying this practice is that the production of ALA is positively correlated with the expression of this gene. However, such strategies are infeasible for examining the relationship between ALA production and the expression profile of each gene in the biosynthetic pathway and their effect on heme production. Due to the structural similarity between ALA, glutamine, and asparagine, it is possible to modify the biosensors of these two amino acids to detect ALA. As previously shown, molecular docking techniques reduce the affinity for arginine and histidine while protecting the sensitivity of lysine through targeted saturation mutagenesis of the screened sites, thereby improving the biosensor sensitivity. Future attempts to modify sensors that respond to ALA analogs for its detection can use similar strategies [84].

### 5.2. Common Heme Detection Systems in Bacteria

**Figure 5:**
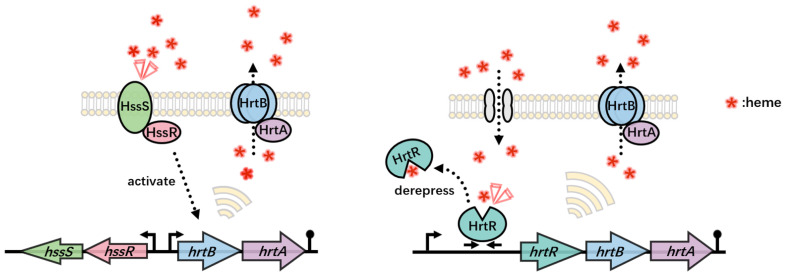
Two different bacterial heme-sensing systems. One outside the cell and one in the cytoplasm, regulating intracellular heme homeostasis through HrtBA. The left panel shows the two-component heme sensing system (HssRS) present in *S. aureus*, where HssS corresponds to heme and is autophosphorylated, while HssR is transphosphorylated, which in turn activates the expression of heme transporter protein (HrtAB). The figure on the right shows the one-component heme sensing system present in *Lactobacillus.* Heme is taken up by the fhuDBA gene product (gray channel) and bound to the available HrtR protein to alleviate the inhibition of the hrtRBA operon. hrtBA activation leads to heme efflux. The red triangle represents activation of the sensor. Red asterisks indicate heme.

There are innate heme-sensing systems in bacteria, which are paired with exocytosis proteins and then used to regulate heme homeostasis in bacteria to maintain their equilibrium. Common heme-sensing systems can be classified into one- and two-component systems (Figure 5).

#### 5.2.1. One-Component Systems

Saillant et al. [85] identified FhtR, a transcriptional regulator in *Enterococcus faecalis* that regulates heme homeostasis by governing an efflux pump named HrtBAEf, which, similar to HrtBA, regulates heme-dependent peroxidase A expression (KatA), thereby alleviating the oxidative stress produced by heme and reducing the toxicity caused by high heme concentrations.

HrtR is a transcriptional regulator in *Lactobacillus lactis* that senses and binds heme as its physiological effector to regulate the expression of the corresponding heme efflux system, stabilizing heme homeostasis [86]. Therefore, this appears to be more conserved in many commensal bacteria than the extracellular heme sensing system (HssRS) used by many Gram-positive bacteria. Separate from *hrtR* expression, *hrtBA* in this system is a potent heme sensor that regulates the response of the heme efflux pump, HrtBA, to maintain nontoxic intracellular heme levels [87].

#### 5.2.2. Two-Component Systems

In *Staphylococcus aureus*, a two-component heme sensing system (HssRS) responds to exposed heme in real time and activates the corresponding heme transporter protein (HrtAB). Notably, in the absence of extracellular heme, endogenous heme synthesized by *S. aureus* is insufficient to activate HssRS, and the efflux protein HrtAB is not activated [88]. Subsequently, Stauff et al. [89] found using multivariate differential gel electrophoresis and mass spectrometry that *S. aureus* growth in high concentrations was linked to the signaling pathway between HssS (sensor histidine kinase) and HssR (response regulator). In addition, heme induction is dependent on direct repetitive DNA sequences within the *hrtAB* promoter [90]. Similarly, in heme-oxygen biosensors, the binding of oxygen to the heme-Fe^2+^ complex in the oxygen-sensing globin domain induces autophosphorylation of its kinase domain through interdomain signal transduction. This type of protein is typically a component of a two-component signal transduction system and is able to respond to oxygen molecules [26,91].

This mechanism between the two-component heme sensing system (HssRS) and the heme detoxification transporter protein (HrtAB) is also present in *Bacillus anthracis* and is used to resist heme toxicity. Moreover, its sensitivity to heme is better, and its function is superior to its evolutionary relative, *Staphylococcus aureus* [92]. Similarly, in *Bacillus thuringiensis*, HssRS directly regulates a manipulator, *hrmXY*, encoding hypothetical membrane proteins, which differs from other strains of Firmicutes characterized by the HssRS and HrtAB systems. Moreover, the HrmXY function is independent of HrtAB and can mitigate heme toxicity differently [93].

### 5.3. Application of Heme Biosensor in Heme Production

As research on the above-mentioned bacterial endohemoglobin sensing systems continues, they are widely used in various aspects of heme elevation in microorganisms.

Establishing a mechanism to control cellular heme supply and demand is at the forefront of the heme production process. In this context, biosensors respond to the metabolite heme, balancing the proper input-output relationship, and are, therefore, responsible for this action. Leung et al. [94] fused ascorbate peroxidase (mAPX), a monomeric heme-binding peroxidase, with mEGFP, a monomeric form of green fluorescent protein, and developed a heme sensor (mAPXmEGFP) to monitor intracellular heme availability. Therefore, this also provides a mechanism for heme-based signaling and modulation.

As discussed in the previous section, “dynamic control” of metabolic pathways can largely enhance the ability of strains to produce high levels of heme. In this process, biosensors are essential for precise host metabolism regulation. Therefore, properties such as sensitivity, affinity, and tunability are crucial for heme production. Many scholars have produced many influential studies in these areas. For instance, Hu et al. [95] developed an optimal biosensor for the heme fine synthesis system in *E. coli* based on the heme-sensing monitoring system of *L. lactis* (HrtR regulator and its binding site *hrtOL*). They improved the sensitivity of HrtR and used it in combination with a small regulatory RNA system for fine-tuning heme levels in *E. coli*. Zhang et al. [96] used mutant biosensors with different binding affinities to influence the regulatory efficiency of the production pathway, thereby increasing ALA yield and successfully enhancing intermediate production, including protoporphyrinogen. Therefore, this offers new possibilities for more dynamic and precise regulation of the heme biosynthesis process and improvement of its yield.

Some other scholars have combined biosensors with other means to achieve greater possibilities. For example, Glanville et al. [28] first constructed a photosensitive pigment heme biosensor using a phytochrome-based fluorophore (PBF) protein (IFP1.4) and bacterial heme oxygenase (HO), then combined it with FACS and massively parallel sequencing to devise a histology-based method for metabolite-coupled transposon sequencing called “Met-Seq.” However, they discovered previously unknown pathways and genes in *Pseudomonas aeruginosa* that may alter heme metabolism flux, identifying 188 genes that may impact intracellular heme levels. Hence, this provides new potential targets for future heme production improvements using metabolic regulation and other methods. In addition, through high-throughput screening, Mike et al. [97] identified VU0038882(882), a highly efficient small molecule activator of HssRS-dependent heme stress response. It is a useful tool for studying bacterial heme biosynthesis and central metabolism. In addition, the evaluation of the effects of possible gene regulation plays a crucial role in the effectiveness and efficiency of improving heme production. In this regard, Ishchuk et al. [98] used the enzyme-bound extension of the yeast 8 model (ecYeast8) to predict the optimal combination of these potential genes. Subsequently, combining a heme ligand binding biosensor (heme-lbb) with a CRISPR-Cas9 toolbox developed for *S. cerevisiae* [99] to incorporate positive gene targets, the computationally determined combinations to enhance heme production were evaluated, resulting in a substantial increase in intracellular heme production.

Although heme-specific biosensors and their use in the efficient production of heme have been explored for many years, the technology is quite mature, and its applications are diverse. However, the design of biosensors targeting heme currently encounters three challenges [2]. First, due to the presence of heme in two oxidation states, namely reduced ferrous Fe^2+^-heme and oxidized ferric Fe^3+^-heme, it is not simple to improve the binding affinity of biosensors for heme. The next challenge is to adjust the probe expression of the heme biosensor to the appropriate level. Finally, consider whether the designed sensor expression will interfere with heme metabolism or other homeostasis and physiological processes. These challenges also indicate, to some extent, the need for future biosensor development efforts.

## 6. Application of Heme Protein and Its Derivatives in Various Fields

Because of its special structure and unique function, heme and heme-containing proteins are widely used in various fields, such as medical, supplements and food processing. The common ones are listed in Table 3.

### 6.1. Medical

The special structure of hemoglobin confers various functions that play a crucial role in the health and efficient functioning of the organism, offering new treatment possibilities for various diseases. By the bioactive gas-carrying capacity of hemoglobin, for example, Kazuaki Taguchi exogenously combined CO with liposomes of hemoglobin capsules to mimic the structure and function of red blood cells to obtain CO-HbV, which significantly inhibited pulmonary fibrosis and had a favorable therapeutic effect on idiopathic pulmonary fibrosis. Wang et al. [109] added hemoglobin, a chelating protein of free hemoglobin, to the storage of free hemoglobin, which could prevent the rapid loss of NO during storage, thereby improving the quality of transfused and stored red blood cells [101]. Moreover, heme proteins play an important role in the biosynthesis and related functions of NO, an important gas molecule in many diseases. The heme iron complex of hemoglobin and myoglobin is capable of producing NO from nitrate and nitrite anions. NO molecule can bind to both the heme-Fe^2+^ complex and the heme-Fe^3+^ complex at the same time, thereby eliminating its own toxicity to some extent [26].

In the external venous blood of mice, rat, and humans, hemoglobin can also react with nitric oxide to form iron nitroso complexes. These complexes can be used to monitor the progression of cardiovascular disease by directly quantifying bioavailable nitric oxide in human circulation using electron paramagnetic resonance spectroscopy. In addition, the results of Zhang et al. [103] showed that the βCys93 subunit in hemoglobin plays a crucial role in cardioprotection. They expanded their analysis of βCys93Ala animals to well-established experimental mouse models of myocardial infarction and cardiomyopathy, exploring the possibility that enhanced transmission of SNO-based vasodilatory activity of the βCys93 subunit in hemoglobin may predict improved oxygenation in heart failure and acute coronary syndromes, as well as tissue oxygenation physiology deficiencies, as a novel therapeutic strategy for other troublesome conditions.

### 6.2. Dietary Supplements

Hemoglobin, an inexpensive and effective source of heme iron, is absorbed by selective heme transporters and is unaffected by metal ions throughout the absorption process [110,111]. Therefore, heme iron is often used as an iron supplement to combat iron deficiency since it is more bioavailability to humans. A common strategy begins with processed foods fortification, such as fortifying staple foods with heme iron. Michael Hoppe et al. [112] attempted to improve iron status in women of childbearing age using a blood-based dietary therapy. The second strategy is to use heme iron directly as a supplement capsule or tablet. For example, a heme-bound iron supplement tablet is commonly used to treat pregnant women with pregnancy-related IDA [8]. In addition, due to the high concentration of heme iron in bovine blood, bovine blood meal contains as much as 195.46 mg/100 g [113]. Bovine blood has been suggested as a low-cost source of heme iron. The heme iron prepared by Ningtang et al. [104] with bovine blood has strong antioxidant activity in vitro, has a good effect on iron deficiency anemia rats, and can be used as an efficient and safe new iron supplement.

### 6.3. Food Processing

Hemoglobin is a rich source of natural red colorants; however, its color is unstable and largely depends on the oxidative state of heme iron. Meat processing stabilizes hemoglobin using various techniques, such as adding the chelating agent nicotinamide to form a complex with heme [105], introducing saturated carbon monoxide (CO) to convert hemoglobin into carboxyhemoglobin, which is resistant to cooking and dehydration [114] or improving packaging.

With the continuous development of the artificial meat industry, the application field of hemoglobin as a colorant has gradually expanded. Impossible Foods Inc. (Redwood City, CA, USA) synthesized the LegHb c2 gene in soybeans and inserted it into *Pichia* yeast, a yeast host. The obtained LegHb protein passed the allergy and toxicity tests and can confer flavor characteristics on animal meat in plant meat products [107]. The direct addition of extracellular heme proteins to the growth medium during cell proliferation and differentiation improves the proliferation and metabolism of bovine skeletal muscle cells during cellular meat production [77]. Therefore, adding hemoglobin during 3D skeletal muscle tissue formation cultured in vitro of cellular meat can give it a more meat-like look and taste [12].

In addition, hemoglobin and nitrite can form nitroso hemoglobin. Additionally, research has demonstrated that the presence of heme promotes nitroso compound formation [100]. Nitrosohemoglobin can also be used as a colorant to improve the color of the emulsified intestine while simultaneously reducing nitrite residue and exerting a strong antioxidant effect. Therefore, it can replace nitrite as a natural pigment in the emulsified intestine, improving sensory qualities. However, nitroso hemoglobin is similar to hemoglobin but very unstable under air and light and easily degrades and fades. Therefore, people use synthesizing glycosylated nitroso hemoglobin method after glycosylation to improve its physical and chemical properties [115]. In 2019, glycosylated nitrosohemoglobin pigment had good solubility under vacuum freeze-drying and 60 ℃ conditions, strong stability in −18 ℃ storage indoor dark light, 15% hydrogen peroxide, and magnesium chloride solution, and was used to preserve pork. Hongtao Zhang et al. [116] added it to pork batter to help form the red color.

Globin has superior emulsifying ability, and Crenwelge et al. [79] found that globin has the best emulsifying ability after comparing it with soy, cottonseed, and milk protein. The emulsifying activity value of globin with an EAI of 28 is much higher than that of soy protein (EAI of 14) and ovalbumin (EAI of 8). Therefore, globin is often added as a food emulsifier in minced meat or sausage preparation. In addition, globin can serve as a fat substitute. In a study conducted by Viana et al. [108], replacing fat with globin and plasma globin reduced the fat content of ham by 25% and 35%, respectively. Adding these fat substitutes increases the moisture and has been observed.

Similarly, PAS (Per-Arnt-Sim) domains are commonly used as signal sites for protein-protein interactions and signal acceptance sites for intermolecular and intramolecular signal transduction [26]. PAS sensors recognize the presence of oxygen and activate genes associated with aerobic metabolism. These heme proteins have different oxygen affinities to suit their functional purposes [29].

## 7. Conclusions

Heme has been widely used in various fields due to its unique structure and indispensable biochemical function. Whole-cell system-based biosynthesis methods are being explored to meet the growing demand for heme.

First, in the past decade, the complex heme generation pathway (especially the newly discovered CPD and SHD pathway) and effector system in microorganisms have been studied, laying the foundation for biosynthesis and expanding research. Secondly, the strategies of efficient heme production by metabolic engineering and other means through various microorganisms as site cells are constantly enriched, and the optimization of culture conditions and technology has greatly improved the yield. In addition, whole-cell-based biosensors and other new technologies in production are no longer limited to high-throughput screening due to synthetic biology and other fields. All these efforts pave the way for further exploration of the efficient production of heme by microorganisms in the future and point out potential new directions.

## Figures and Tables

**Table 3:** Application of Heme Protein and its Derivatives in Various Fields.

Field	Categories	Application	Ref
Medical	Heme	Catalyzes the formation of nitroso-compounds	[100]
	Hemoglobin	Biomimetic CO delivery system based on hemoglobin	[101]
		Quantitative monitoring of nitric oxide	[102]
	Hemoglobin βCys93	Cardioprotection	[103]
	Globin	Red blood cells storage	[101]
Dietary supplements	Heme iron	Iron supplement	[104]
Food processing	Hemoglobin	Natural color enhancer	[105]
		Coloration of cell-based meat.	[12]
	Nitrosohemoglobin	Coloration of meat	[106]
	Glycosylated nitrosohemoglobin	Coloration of meat	[106]
	Soy leghemoglobin	Increase meat-like flavor of plant-based meat	[107]
	Globin	Emulsifier	[79]
		Fat substitute	[108]

## Data Availability

We didn’t create any new date.

## Abbreviations

Abbreviations and their full names are listed in here for your convenience. List of Names and Abbreviations in Whole Article.

**Abbreviation**	**Definition**
AlaS	ALA synthase
ALA	5-aminolevulinic acid
CBM	cell-based meat
CgdC	Coproporphyrinogen decarboxylase
CgdH	Coproporphyrinogen dehydrogenase
CgoX	Coproporphyrinogen oxidase
ChdC	Coproheme decarboxylase
ChdH	Coproheme dehydrogenase
CPIII	Coproporphyrinogen III
CPD	Coproporphyrin-dependent
CpfC	Coproporphyrin ferrochelatase
CoA	Coenzyme A
DBTL	Design-build-test-learning
DtxR	Diphtheria toxin repressor
DyP	Dye-decolorizing peroxidase
FACS	Fluorescence-activated cell sorting
GluRS	Glutamyl-tRNA synthetase
GsaM	Glutamate-1-semialdehyde-2,1-aminomutase
GtrR	Glutamyl-tRNA reductase
HmbS	Hydroxymethylbilane synthase
HO	Heme oxygenase
HPLC	High-Performance Liquid Chromatography
IDA	Iron deficiency anemia
MAE	Malic enzyme
NGS	Next-generation sequencing
PANK	Pantothenate kinase
PBF	Phytochrome-based fluorophore
PbgS	Porphobilinogen synthase
PBM	Plant-based meat
SfGFP	Super folder green fluorescent protein
TCA	Tricarboxylic acid
PAS	Per-Arnt-Sim
PcdH	Precorrin-2 dehydrogenase
PcyA	Phycocyanobilin: ferredoxin oxidoreductase
PgdH1	Protoporphyrinogen dehydrogenase
PgdH2	Protoporphyrinogen dehydrogenase
PgoX	Protoporphyrinogen IX oxidase
PPD	Protoporphyrin-dependent
PpfC	Protoporphyrin ferrochelatase
PPIX	Protoporphyrin IX
SHD	Siroheme-dependent
ShfC	Sirohydrochlorin ferrochelatase
SumT	S-adenosyl-L-methionine dependent uroporphyrinogen methyltransferase
TRNA	Transfer RNA
UroD	Uroporphyrinogen decarboxylase
UroS	Uroporphyrinogen synthase
